# Somatic alterations in circulating cell-free DNA of oesophageal carcinoma patients during primary staging are indicative for post-surgical tumour recurrence

**DOI:** 10.1038/s41598-018-33027-4

**Published:** 2018-10-08

**Authors:** Helen Pasternack, Jana Fassunke, Patrick Sven Plum, Seung-Hun Chon, Daniel Alexander Hescheler, Asmae Gassa, Sabine Merkelbach-Bruse, Christiane Josephine Bruns, Sven Perner, Michael Hallek, Reinhard Büttner, Elfriede Bollschweiler, Arnulf Heinrich Hölscher, Alexander Quaas, Thomas Zander, Jonathan Weiss, Hakan Alakus

**Affiliations:** 1 Institute of Pathology, University Medical Center Schleswig-Holstein, Campus Luebeck and Research Center Borstel, Leibniz Lung Center, Luebeck and Borstel, Germany; 2 Institute of Pathology, University Hospital Cologne, Cologne, Germany; 3 Department of General, Visceral and Cancer Surgery, University Hospital Cologne, Cologne, Germany; 4 Department of Cardiothoracic Surgery, University Hospital of Cologne, Cologne, Germany; 5 Department of Internal Medicine I, University Hospital Cologne, Cologne, Germany; 6 Center for Oesophageal and Gastric Surgery, AGAPLESION Markus Hospital, Frankfurt am Main, Germany; 7 Gastrointestinal Cancer Group Cologne, Cologne, Germany

## Abstract

Oesophageal cancer (OC) has high mortality. This study aims at determining the feasibility of liquid biopsies for genomic profiling in early stage OC, comparing two different technologies for mutational analysis in circulating cell -free DNA (ccfDNA) and evaluating the clinical impact of these somatic alterations during primary staging. In 25 patients with locally advanced OC, endoscopic tumour biopsies and simultaneous blood samples were taken during primary staging. Genomic DNA from biopsies and ccfDNA were analysed for mutations using a 12 gene panel next-generation sequencing (NGS) assay as well as digital droplet PCR (ddPCR). Genetic data was correlated with patients’ outcome. In 21 of the tested biopsies (84%) at least one somatic mutation was detected by NGS. Mutations detected by NGS were detectable by ddPCR with similar allele frequencies. In three out of the 21 patients with proven mutations, the same mutations were also detectable in ccfDNA using NGS (14%). In contrast, ddPCR detected mutations in ccfDNA of five additional patients (8/21, 38%). Post-surgical outcome analysis was performed for those patients who had received complete tumour resection (n = 16). Five of them suffered from an early relapse within the first year after surgery, including four with detectable somatic mutations in ccfDNA during primary staging. Taken together, we showed a higher sensitivity for ddPCR compared to NGS in detecting mutated ccfDNA in OC. Detection of somatically altered ccfDNA during primary staging seems to be indicative for post-surgical tumour recurrence.

## Introduction

Oesophageal cancer (OC) ranks among the most common malignancies, with an increasing incidence rate world-wide. Especially oesophageal adenocarcinoma spreads rapidly throughout the western countries. The main treatment options for OC are surgical resection and radio-chemotherapy and although treatment has advanced (mostly by better management of treatment related side effects) in the last years, patients’ outcomes are still poor, with a five-year survival rate of less than 20%^1^. Unfortunate outcomes are mainly due to late detection of primary as well as recurrent tumours. Therefore, improvement of early tumour detection is essential in order to achieve better cure rates in OC.

Analysis of somatic alterations in tumour tissue has become routine practice in clinical oncology. Although these alterations are highly informative, sampling tumour tissue has limitations as tissue biopsies are often difficult to obtain and are subjected to sampling bias resulting from temporal and spatial tumour heterogeneity. Therefore, alternative strategies, such as liquid biopsies, are currently evaluated for applicability in different clinical settings. Liquid biopsies represent a non-invasive diagnostic tool, generally defined as a diagnostic procedure in which information on a tumour disease is gained from body fluids. Especially, the analysis of circulating cell-free DNA (ccfDNA) has gained more and more attention recently. The presence of ccfDNA in human blood was already described in 1948^2^. Since then multiple studies have demonstrated that in cancer patients ccfDNA levels are increased compared to healthy individuals and that somatic mutations corresponding to tumour derived DNA can be detected in the patients’ plasma^3–8^. As the fraction of ccfDNA derived from tumours, also known as circulating tumour DNA (ctDNA), is often extremely low, the detection of somatic mutations in ccfDNA remained a diagnostic challenge for a long time^9,10^. With the development of more sensitive mutational analysis techniques, such as next-generation sequencing or digital droplet PCR, the detection of ccfDNA carrying somatic mutations has now become clinically feasible^5,11^. The mutational analysis of ccfDNA supplements the histological diagnosis of cancer with providing additional valuable information on disease progression, resistance to targeted therapy or tumour heterogeneity.

Although there are a lot of studies dealing with the analysis of ccfDNA in many different solid tumour entities, so far only a few have investigated oesophageal carcinomas. Furthermore, there is a lack of studies focusing on localised disease with low tumour burden. Herein, we therefore report on the feasibility of using liquid biopsies for genomic profiling of OC at the early time of primary staging. We compare next-generation sequencing and digital droplet PCR as two different technologies for mutational analysis of ccfDNA. Furthermore, we evaluate the clinical impact of detectable somatic alterations in ccfDNA during primary staging.

## Results

### Patient characteristics and study design

**Figure 1:**
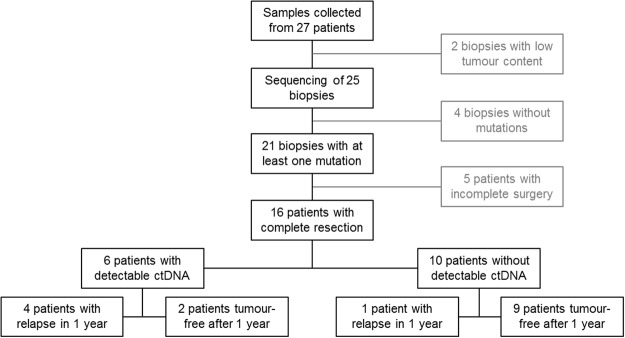
Study Workflow showing respective sample numbers for each analysis step. Methodical comparison of next-generation sequencing vs. digital droplet PCR for the detection of tumour DNA in plasma is performed on data of 21 patients with identified mutations. Clinical outcome analysis is performed for 16 patients as five patients had to be excluded due to incomplete surgery.

27 patients with oesophageal cancer (OC) were included in this study (see Table 1 and Fig. 1). The patient cohort comprised 7 female and 20 male individuals, median age was 65 years. One patient presented with an early local tumour (T1), all other patients had locally advanced tumours (T2–3). Most patients underwent surgical tumour resection (21/27) with or without neoadjuvant radio-chemotherapy. In six cases no surgery was carried out due to the patients’ functional situation or individual wish. More detailed information on performed surgery and pathological staging of resected tumours are shown in Supplemental Table S1. Five of the patients suffered from an early post-surgical relapse within the first 12 months. Tumour recurrence resulted in tumour related death in two of these patients.

**Table 1:** Patient cohort. Within the current study patients with locally advanced oesophageal cancer of both histopathological subtypes (adenocarcinoma and squamous cell carcinoma) were included.

Case	Age [years]	Sex	Histology	Primary staging	Resection	Relapse within 1 year after resection	Time to relapse [years]	TRD
1	79	f	adeno	uT2 N+	yes	no	n.a.	no
2	82	m	adeno	uT3 N+	no	n.a. (no resection)	n.a.	yes
3	56	m	adeno	uT3 N+	yes	no	n.a.	no
4	56	f	adeno	uT3 N+	yes	no	n.a.	no
5	62	m	squamous	uT3 N+	no	n.a. (no resection)	n.a.	no
6	57	f	adeno (signet ring cell)	uT1 Nx	yes	no	n.a.	no
7	73	m	squamous	cT3 N+	no	n.a. (no resection)	n.a.	no
8	75	f	adeno (signet ring cell)	uT3 N+	yes	no	n.a.	no
9	52	m	Barret	uTx Nx	yes	no data available	n.a.	no
10	65	m	adeno	uT3 N+	no	n.a. (no resection)	n.a.	no
11	71	m	adeno	uT3 N+	yes	yes, cutanous	0.8	yes
12	59	f	squamous	uT3 Nx	no	n.a. (no resection)	n.a.	no
13	76	m	adeno	uT3 N+	yes	no	n.a.	no
14	75	m	adeno	uT3 N+	yes	yes, retroperitoneal lymph nodes	0.8	no
15	52	f	squamous	uT3 Nx	yes	no	n.a.	no
16	56	m	adeno	uT3 Nx	yes	no	n.a.	no
17	71	m	adeno	uT3 N+	yes	no	n.a.	no
18	83	f	adeno	uT3 N+	yes	no	n.a.	no
19	58	m	squamous	uT2 Nx	yes	no	n.a.	no
20	47	m	adeno	uT3 Nx	yes	no	n.a.	no
21	73	m	andeno	uT3 N+	yes	yes, proximal to anastomosis	0.4	no
22	80	m	adeno	uT3 N+	yes	yes, meningeosis carcinomatosa/osseous	0.5	yes
23	70	m	adeno	uT3 Nx	yes	no	n.a.	no
24	64	m	squamous	uT3 Nx	no	no	n.a.	no
25	77	m	squamous	uT3 N+	yes	no	n.a.	no
26	56	m	adeno	uT2 Nx	yes	no	n.a.	no
27	52	m	adeno	uT3 Nx	yes	yes, adrenal gland	0.2	no

The patients’ blood was sampled during endoscopy in primary staging. Most patients underwent surgical resection with/without neoadjuvant radio-chemotherapy. In six cases no surgery was carried out due to the patients’ functional situation or individual wish.

TRD: tumour related death, m: male, f: female, n.a.: not applicable.

Peripheral blood was collected simultaneously with routine endoscopic tumour biopsies at the time of primary staging.

### Mutational analysis of gDNA from tumour biopsies

**Figure 2:**
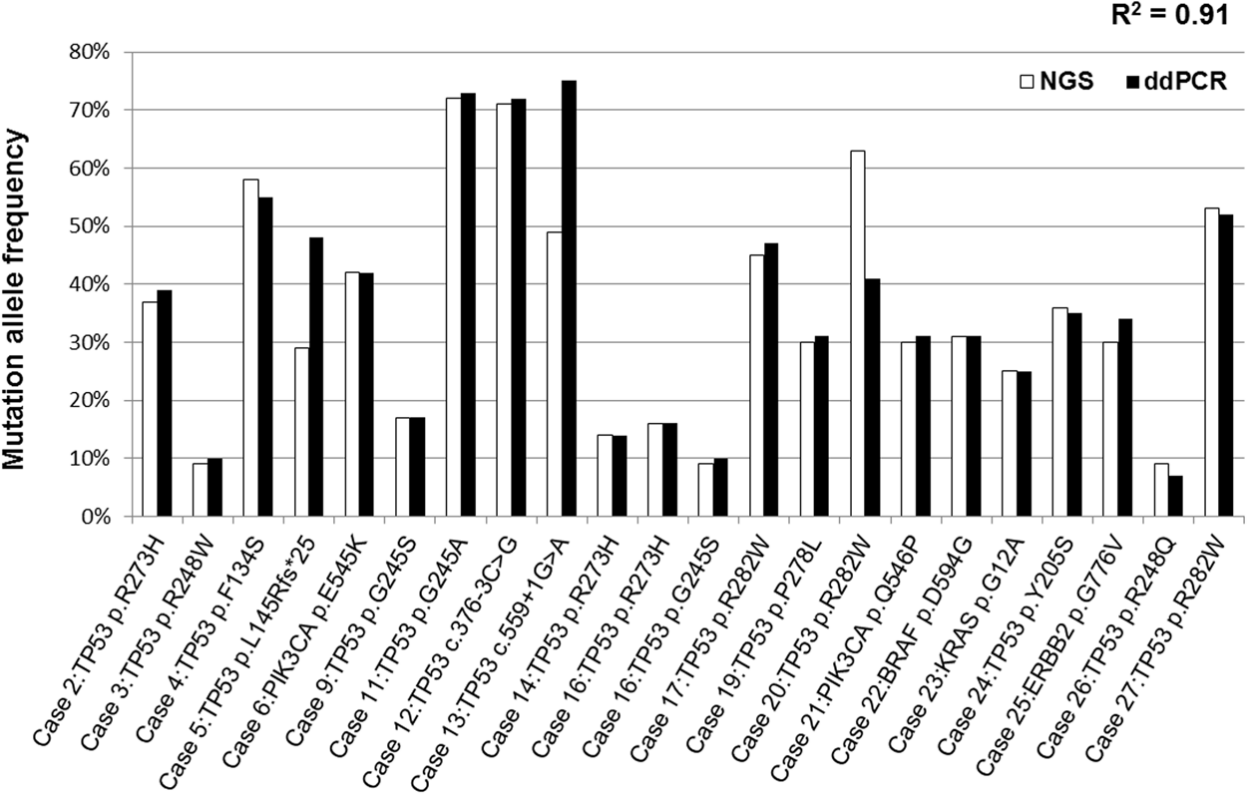
Comparison of mutation allele frequencies by next-generation sequencing (white bars) and digital droplet PCR (black bars) on genomic DNA. The Pearson approach was used to determine the correlation between the two datasets (R^2^ = 0.91).

From all biopsies with at least 10% tumour cell content (25/27) genomic DNA (gDNA) was extracted and analysed by means of targeted next-generation sequencing using a custom gene panel. The tumour content in the remaining samples (case 10 and 15) was too low (see Table 2). Therefore, these samples were excluded from further analysis (see Fig. 1). The gene panel used was designed for genomic analysis of gastrointestinal tumours and comprised amplicons of relevant DNA regions from 12 genes including *BRAF, DDR2, ERBB2, HRAS, KEAP1,KRAS, NFE2L2,NRAS,PIK3CA, PTEN, RHOA* and *TP53* (see Supplemental Table S2). In 84% of the analysed biopsies (21/25) at least one somatic mutation could be detected within the 12 gene panel. Cases 6, 16, 22, 23 and 24 harboured two mutations respectively and the majority of mutations detected were located in the *TP53* gene. Determined allele frequencies ranged from 9% to 72%, an overview of the detected mutations is shown in Table 2. For every patient with identified mutations at least one mutation detected with next-generation sequencing (NGS) was additionally validated by digital droplet PCR (ddPCR), using pre-designed and custom made assays. Mutations were detected by both methods in very similar allele frequencies (see Fig. 2). Only for three mutations the detected allele frequencies differed from each other by more than 4% (cases 5, 10, 16). The consistency between both datasets was further determined by Pearson’s correlation analysis which showed an R² of 0.91.

**Table 2:** Detectable somatic mutations in circulating cell-free DNA (ccfDNA) from blood plasma compared to genomic DNA (gDNA) derived from the surgical tumour specimens utilising next-generation sequencing with a panel of 12 genes including *BRAF, DDR2, ERBB2, HRAS, KEAP1, KRAS, NFE2L2, NRAS, PIK3CA, PTEN, RHOA* and *TP53* as well as digital droplet PCR. Measured allele frequencies are shown in %.

Case	estimated tumour cell content	mutational analysis gDNA		mutational analysis ccfDNA	
		NGS	ddPCR	NGS	ddPCR
1	40%	wildtype	not analysed	wildtype	not analysed
2	80%	TP53: c.818G > A p.R273H 37%	TP53: c.818G > A p.R273H 39%	wildtype	wildtype
3	10%	TP53: c.742C > T p.R248W 9%	TP53: c.742C > T p.R248W 10%	wildtype	wildtype
4	90%	TP53: c.401T > C p.F134S 58%	TP53: c.401T > C p.F134S 55%	wildtype	wildtype
5	70%	TP53: c.434delT p.L145Rfs*25 29%	TP53: c.434delT p.L145Rfs*25 48%	TP53: c.434delT p.L145Rfs*25 1%	TP53: c.434delT p.L145Rfs*25 1.1%
6	90%	RHOA: c.205C > G p.L69V 33%;	RHOA: not analysed;	wildtype	wildtype
		PIK3CA: c.1633G > A p.E545K 42%	PIK3CA: c.1633G> Ap.E545K 42%		
7	10%	wildtype	not analysed	wildtype	not analysed
8	10%	wildtype	not analysed	wildtype	not analysed
9	10%	TP53: c.733G > A p.G245S 17%	TP53: c.733G > A p.G245S 17%	wildtype	wildtype
10	2%	not analysed (low tumour content)		wildtype	not analysed
11	90%	TP53: c.734G > C p.G245A 72%	TP53: c.734G > C p.G245A 73%	TP53: c.734G > C p.G245A 1%	TP53: c.734G > C p.G245A 0.22%
12	80%	TP53: c.376–3C > G 71%	TP53: c.376–3C > G 72%	wildtype	TP53: c.376–3C > G 1.4%
13	80%	TP53: c.559 + 1G > A 49%	TP53: c.559 + 1G > A 75%	wildtype	wildtype
14	60%	TP53: c.818G > A p.R273H 14%	TP53: c.818G > A p.R273H 14%	wildtype	TP53: c.818G > A p.R273H 1.4%
15	2%	not analysed (low tumour content)		wildtype	not analysed
16	20%	TP53: c.818G > A p.R273H 16%; TP53: c.733G > A p.G245S 9%	TP53: c.818G > A p.R273H 16%; TP53: c.733G > A p.G245S 10%	wildtype	wildtype
17	90%	TP53: c.844C > T p.R282W 45%	TP53: c.844C > T p.R282W 47%	wildtype	TP53: c.844C > T p.R282W 0.25%
18	70%	wildtype	not analysed	wildtype	not analysed
19	30%	TP53: c.833C > T p.P278L 30%	TP53: c.833C > T p.P278L 31%	wildtype	wildtype
20	80%	TP53: c.844C > T p.R282W 63%	TP53: c.844C > T p.R282W 41%	wildtype	wildtype
21	90%	PIK3CA: c.1637A > C p.Q546P 30%	PIK3CA: c.1637A > C p.Q546P 31%	wildtype	PIK3CA: c.1637A > C p.Q546P 0.5%
22	40%	BRAF: c.1781A > G p.D594G 31%; TP53: c.614 A > T p.Y205F 35%	BRAF: c.1781A > G p.D594G 31%; TP53: not analysed	wildtype	wildtype
23	50%	KRAS: c.35G > C p.G12A 25%; KRAS: c.347A > G p.N116S 25%; PTEN: c.79 T > A p.Y27N 36%	KRAS: c.35G > C p.G12A 25%; KRAS: c.347A > G not analysed; PTEN: not analysed	wildtype	wildtype
24	70%	TP53: c.614A > C p.Y205S 36%; TP53: c.880G > T p.E294* 25%	TP53: c.614A > C p.Y205S 35%; TP53: c.880G > T not analysed	wildtype	wildtype
25	80%	ERBB2: c.2327G > T p.G776V 30%	ERBB2: c.2327G > T p.G776V 34%	wildtype	ERBB2: c.2327G > T p.G776V 0.2%
26	60%	TP53: c.743G > A p.R248Q 9%	TP53: c.743G > A p.R248Q 7%	wildtype	wildtype
27	80%	TP53: c.844C > T p.R282W 53%	TP53: c.844C > T p.R282W 52%	TP53: c.844C > T p.R282W 2.4%	TP53: c.844C > T p.R282W 3.5%
**Positive Control:**					
M1	10% (in 2014)	PIK3CA: c.1624G > A p.E542K 5%	PIK3CA: c.1624G > A p.E542K 20%	PIK3CA: c.1624G > A p.E542K 14%	PIK3CA: c.1624G > A p.E542K 6%

gDNA: genomic DNA, ccfDNA: circulating cell-free DNA, NGS: next-generation sequencing, ddPCR: digital droplet PCR.

### Mutational analysis of ccfDNA from plasma samples

**Figure 3:**
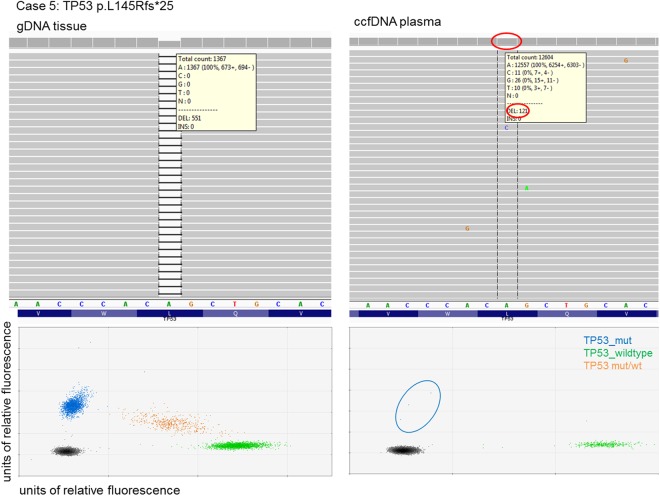
Visual representation of a single mutation in gDNA and ccfDNA. The upper panels show results from next-generation sequencing, whereas the lower panels show the same DNA analysed by digital PCR. The left panels show the mutation on genomic DNA, the right panels show the results from corresponding ccfDNA.

From all 27 patients peripheral blood samples were obtained and in each case plasma was extracted immediately after blood collection. Circulating cell-free DNA (ccfDNA) was extracted and analysed with the same 12 gene panel as described for tumour biopsies using NGS. In Table 2 the obtained results from the performed mutational analyses are given. From the 21 cases with somatic mutations identified in tissue based analysis only in three cases the respective mutation could also be detected in ccfDNA (14%). Furthermore, of these three mutations only two were primarily called by the used bioinformatics analysis pipeline (*TP53*: p.L145Rfs*25, case 5 and *TP53*: p.R282W, case 27) whereas the other mutation (*TP53*: p.G245A, case 11) could only be detected by manual inspection using a genome viewer with a focus particular on the mutated sequence site. The determined allele frequencies in ccfDNA samples accounted for 1% to 2.4% indicating a low fraction of tumour derived DNA (compare Fig. 3, upper panels).

All analysed cases showed very low amounts of ccfDNA in the plasma (on average 3 ng per ml plasma, data not shown). Due to the limited DNA input volume for the NGS assay we could only use ~1 ng DNA. Such low amounts of input DNA were strongly limiting the sensitivity of the NGS assay and probably reflect the low tumour burden of the patients. To ensure our NGS pipeline is able to detect mutations in ccfDNA, we also analysed a plasma sample from an OC patient suffering from metastatic disease (case M1) as a positive control. In this case an activating *PIK3CA* p.E542K mutation was known from tissue based analysis performed in routine molecular diagnostics. In contrast to the analysed ccfDNA samples from patients with locally advanced tumours this ccfDNA showed the same mutation as the corresponding tumour tissue in a quite high and well detectable allele frequency of 14% (see Table 2 and Supplemental Figure SF1).

As mutation detection rate in ccfDNA using NGS was limited we further applied ddPCR for its’ very low limit of detection (see Fig. 3, lower panels). Therefore, the 21 cases with identified mutations were analysed with ddPCR (see Supplemental Table S3). Using ddPCR mutations in ccfDNA in the same three plasma samples as with NGS (cases 5, 11, 27) were detected. In addition, ddPCR identified somatic alterations in ccfDNA of five more patients (cases 12, 14, 17, 21, 25, Table 2). Thus, using ddPCR for mutational analysis of ccfDNA a higher detection rate could be achieved than with NGS (8/21, 38% vs. 3/21, 14%). The determined allele frequencies by ddPCR analysis were similar to those in NGS based analysis and below 1.5% in most cases.

### Correlation of results from mutational analysis with patients’ outcome

**Figure 4:**
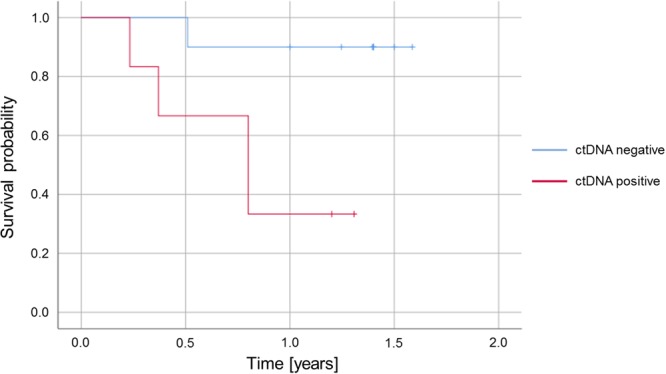
Kaplan-Meier plot representing the probability to relapse within 1 year for patients who underwent complete resection and who had mutations detectable in tissue-based analysis (n = 16). Compared are patients with and without detectable levels of circulating tumour DNA (ctDNA) at primary staging.

Of the 27 patients analysed in this study 21 received complete tumour resection and were evaluated for post-surgical tumour recurrence within the considered follow-up period of at least 12 months. Of these patients 16 had identified mutations in tissue-based analysis und could therefore be evaluated regarding the detection of somatic mutations in ccfDNA (see Fig. 1). Progression free survival was shorter for patients with detectable somatic alterations in ccfDNA during primary staging (see Fig. 4). 67% of the patients with detectable somatic alterations in ccfDNA during primary staging developed an early relapse (4/6, see Tables 1 and 2). In contrast, only one of the ten patients lacking detectable mutations in ccfDNA at the time of primary staging suffered from tumour recurrence within the considered follow-up period (1/10, 10%). These data indicate that detection of somatic alterations in the ccfDNA from patients with locally advanced OC predicts tumour reoccurrence post-surgery (p = 0.036, Fisher’s exact test).

## Discussion

Oesophageal cancer is often characterised by unfortunate outcomes mainly due to late detection of primary as well as recurrent tumours. Therefore, we evaluated the feasibility of using the presence of somatic mutations in ccfDNA at the time of primary staging in addition to endoscopic biopsies as a biomarker for post-surgical outcome of OC patients. From patients with locally advanced OC routine endoscopic tumour biopsies and simultaneous blood samples were taken at the time of primary staging. In 84% (21/25) of the analysed biopsies at least one somatic mutation could be detected with the 12 gene NGS panel used. The high detection rate clearly demonstrates that the targeted sequencing panel, although only comprising a limited number of genes, is highly suitable for genomic profiling of OC. In line with the known mutational landscape of OC we observed a high proportion of *TP53* mutations in our cohort of both adeno and squamous cell histology tumours^12–15^. Corresponding to the notably different tumour cell contents in the analysed biopsies, the determined allele frequencies varied greatly and ranged from 9% to 72%. The allele frequencies obtained from NGS based analysis were in high concordance with those detected by ddPCR confirming the accuracy and robustness of both methods. For mutation detection in circulating cell-free DNA, sample preparation is a key factor. Plasma must be prepared within a 4 hour window after blood draw, otherwise the sample might be compromised by wildtype DNA released by leukocytes^5,16^. Therefore, in this study all blood samples were processed directly after collection. In the detection of somatic mutations from ccfDNA, ddPCR reached a higher detection rate compared to NGS (8/21, 38% vs. 3/21, 14%) reflecting the superior sensitivity of this method. Despite its high sensitivity ddPCR has an inherent limitation compared to NGS because of the prerequisite that the considered mutations have to be known exactly in advance. Therefore, ddPCR will be the method of choice whenever one or more mutations of a tumour are already known, for example from tissue based analysis whereas for primary genomic profiling methods based on multi gene panels, just as NGS, are more applicable. The overall low tumour DNA detection rate in ccfDNA with both methods is most likely due to the early stage disease and therefore low tumour burden and low amounts of tumour DNA in the blood of the patients involved in this study. A similar correlation has already been described in different tumour entities like lung and colorectal cancer^16–18^. This circumstance is further underlined by the low amount of ccfDNA in our cohort showing ccfDNA levels elevated only slightly or even not at all compared to healthy individuals^5^. Furthermore, the fraction of tumour derived DNA in these small amounts of ccfDNA are completely unknown and might also be only minor. Therefore, a metastatic stage tumour was included in the study as a positive control. As expected, in this case detection of a somatic *PIK3CA* mutation known from tissue based analysis was easily feasible in ccfDNA.

Despite the overall low mutation detection rates in the analysed ccfDNA samples the detection of somatic mutations in the ccfDNA of OC patients at the early time of primary staging was strikingly associated with post-surgical tumour recurrence in our cohort. Post-surgical outcome could be analysed for 16 patients with identified mutations and complete resection. From six patients with detectable somatic alterations in ccfDNA four suffered from an early relapse (67%) whereas of ten patients without detected mutations in ccfDNA only one presented with tumour recurrence in the first year after surgery (10%). Other studies on the analysis of ccfDNA in OC so far focused on the time period after tumour resection and on squamous cell histology. Hsieh *et al*. could show that higher ccfDNA levels after oesophagectomy are associated with poorer disease free survival of oesophageal squamous cell carcinoma patients^19^. Similar results were obtained by Ueda *et al*., who describe the usage of somatic mutations in ccfDNA as a biomarker for post-surgical oesophageal squamous cell carcinoma recurrence^20^. The application of mutational analysis on ccfDNA for monitoring treatment effects and early detection of oesophageal squamous cell carcinoma was also depicted by Luo *et al*.^21^.

The present study demonstrates a significant predictive value of somatic alterations in ccfDNA at primary staging for development of an early post-surgical tumour relapse. Thus, screening for somatic mutations in the ccfDNA of OC patients already at the time of primary staging might be clinically relevant in indicating a higher risk for post-surgical tumour recurrence. Due to the individual design of each ddPCR assay also mutational analysis of the patients’ tumour tissue would have to be performed in parallel in order to define the specific sequence to be analysed. ddPCR was clearly superior in the low frequency mutation detection in ccfDNA compared to NGS and would thus be the method of choice in this context. Additional larger studies will be required to confirm the feasibility of using the detection of somatic mutations in ccfDNA at the time of primary staging as a biomarker for post-surgical outcome of OC patients.

## Methods

### Patients

27 patients with either oesophageal adenocarcinoma or squamous cell carcinoma undergoing routine staging including esophagogastroduodenoscopy, endoscopic ultrasound as well as spiral contrast enhanced computer tomography of thorax and abdomen participated in the analysis. During routine endoscopy, tumour biopsies as well as peripheral blood samples were taken and processed as described later. Depending on the clinical stage of tumour disease, patients either underwent primary oesophagectomy (early local tumour such as cT1) or received a neoadjuvant radio-chemotherapy analogue the CROSS-protocol followed by surgery (locally advanced tumours such as uT2–3)^22^. Another therapeutic option was a definitive radio-chemotherapy if the patients’ functional situation or disseminated metastasis did not allow a surgical approach. Standard surgical procedure was laparotomic or laparoscopic gastrolysis and right transthoracic en-bloc oesophagectomy including two-field lymphadenectomy of mediastinal and abdominal lymph nodes. Reconstruction was performed by high intrathoracic esophagogastrostomy as described previously^23^. Prognosis and histopathological outcome were analysed retrospectively. Follow-up time was at least 12 months for each case. The study was approved by the Institutional Ethics Committee of the University Hospital of Cologne (Ethics-No. 13–091, BioMaSOTA) and written informed consent was obtained from all patients before enrolment into the study. All experiments were performed in accordance with relevant guidelines and regulations.

### Histopathology on resected tumours

Resulting oesophageal specimens as well as resected lymph nodes were fixed within 5% formaldehyde and completely embedded in paraffin. Afterwards, 2–3 µm thick slides were cut and stained using haematoxylin and eosin. Further staining with periodic acid/Schiff of the oesophagus specimens were performed if necessary for better evaluation of the depth of tumour infiltration. All surgical specimens were classified by experienced gastrointestinal pathologists according to the seventh edition of the Union for International Cancer Control/TNM-classification of malignant tumour.

### Blood sampling and preparation of plasma

From all patients 20 ml EDTA blood samples were taken at the time of primary staging. Plasma was isolated from whole blood by centrifugation at 3000 rpm for 10 minutes at 4 °C in a 50 ml collection tube. The supernatant was subsequently centrifuged at high speed (at 16,000 g, 10 min., 4 °C) and transferred to a fresh low-binding DNA tube (Eppendorf AG). The samples were stored at −80 °C. Before ccfDNA extraction, the samples were equilibrated to room temperature (15–25 °C).

### DNA extraction

Extraction of circulating cell-free DNA was carried out with 4 ml plasma with the QIAsymphony device (QIAGEN) using the QIAsymphony PAXgene Blood ccfDNA kit according to the manufacturer’s protocol. The genomic DNA extraction from fresh frozen biopsy sections was carried out with the QIAamp DNA Mini kit (QIAGEN) according to the manufacturer’s protocol.

### Next-generation sequencing

Mutational analysis was performed by next-generation sequencing (NGS) using an Ion AmpliSeq Custom DNA Panel (Thermo Fisher Scientific) and the Ion AmpliSeq Library Kit 2.0 (Thermo Fisher Scientific) according to the Ion AmpliSeq Library Preparation User Guide (Thermo Fisher Scientific). After multiplex PCR, libraries were generated by adapter ligation and target enrichment using the Gene Read DNA Library I Core Kit, the Gene Read DNA I Amp Kit (QIAGEN) and the NEXTflex DNA Barcodes (Bio Scientific). 12 pM of the constructed libraries were sequenced on the MiSeq platform (Illumina) with a MiSeq reagent kit V2 (Illumina) with 300 cycles following the manufacturer’s recommendations. Data analysis and mutation calling were performed as previously described^24^.

### Digital droplet PCR

In digital droplet PCR (ddPCR), a PCR reaction is split into ~ 20000 individual droplets, each containing, in average, zero or one DNA molecule. During the readout, each droplet is individually counted and accessed for fluorescence. All mutation detection probes were supplied by Bio-Rad and order numbers are listed in Supplemental Table S3. In each experiment, the number of wildtype DNA molecules was determined in the same reaction using a second probe complementary to the wildtype sequence of the tested gene. Amplifications were carried out in a reaction volume of 20 µL on the QX200 Droplet Digital PCR System (Bio-Rad). Each PCR reaction contained 3 µL PCR grade water, 10 µL Bio-Rad PCR mix for Probes, 1 µL of each (target and reference) amplification primer/probe mix. In case of genomic DNA, 1 µL of DNA and 4 µL of PCR grade water were added to bring the PCR reaction to a 20 µL total volume. Due to the low concentration of ccfDNA, 5 µL were added to the PCR reaction. PCR cycling was performed on a C1000 thermo cycler (Bio-Rad) according to manufacturer’s instructions. Results were analysed with Quantasoft v.1.3.2 software (Bio-Rad) and reported as allelic frequencies.

### Statistical analyses

Statistical analysis was performed in R (Version 3.3.1) using the standard statistics package. Kaplan-Meier calculations were performed using SPSS. Correlation between NGS and ddPCR allele frequencies was performed using the Pearson approach. Association of an early post-surgical relapse with detection of somatic mutations in ccfDNA at primary staging was checked with Fisher’s exact test.

## Electronic supplementary material

Dataset 1

## Data Availability

The datasets generated during and/or analysed during the current study are available from the corresponding author on reasonable request.
