# Supplementary material for: Somatic alterations in circulating cell-free DNA of oesophageal carcinoma patients during primary staging are indicative for post-surgical tumour recurrence
Source: Sci Rep. 2018 Oct 8;8:14941. doi: 10.1038/s41598-018-33027-4 (PMC6175817; doi:10.1038/s41598-018-33027-4)
Supplement: Supplementary file 1 — Dataset 1 [file 41598_2018_33027_MOESM1_ESM.docx]

**SUPPLEMENTAL INFORMATION**

**Somatic alterations in circulating cell-free DNA of oesophageal carcinoma patients during primary staging are indicative for post-surgical tumour recurrence**

Helen Pasternack^1,+^, Jana Fassunke^2,+^, Patrick Sven Plum^3^, Seung-Hun Chon^3^, Daniel Hescheler^3^, Asma Gassae^4^, Sabine Merkelbach-Bruse^2^, Christiane Bruns^3^, Sven Perner^1^, Michael Hallek^4^, Reinhard Büttner^2^, Elfriede Bollschweiler^3^, Arnulf Heinrich Hölscher^6^, Alexander Quaas^2^, Thomas Zander^5,#^,Jonathan Weiss^5,#^, Hakan Alakus^3,#*^

^1^Pathology of the University Medical Centre Schleswig-Holstein, Campus Luebeck and Research Centre Borstel, Leibniz Lung Centre, Luebeck and Borstel, Germany

^2^Institute of Pathology, University Hospital Cologne, Cologne, Germany

^3^Department of General, Visceral and Cancer Surgery, University Hospital Cologne, Cologne, Germany

^4^ Department of Cardiothoracic Surgery, University Hospital of Cologne, Cologne, Germany

^5^Department of Internal Medicine I, University Hospital Cologne, Cologne, Germany

^6^Center for Oesophageal and Gastric Surgery, AGAPLESION Markus Hospital, Frankfurt am Main, Germany

*** Corresponding Author: hakan.alakus@uk-koeln.de**

^+, #^Authors contributed equally


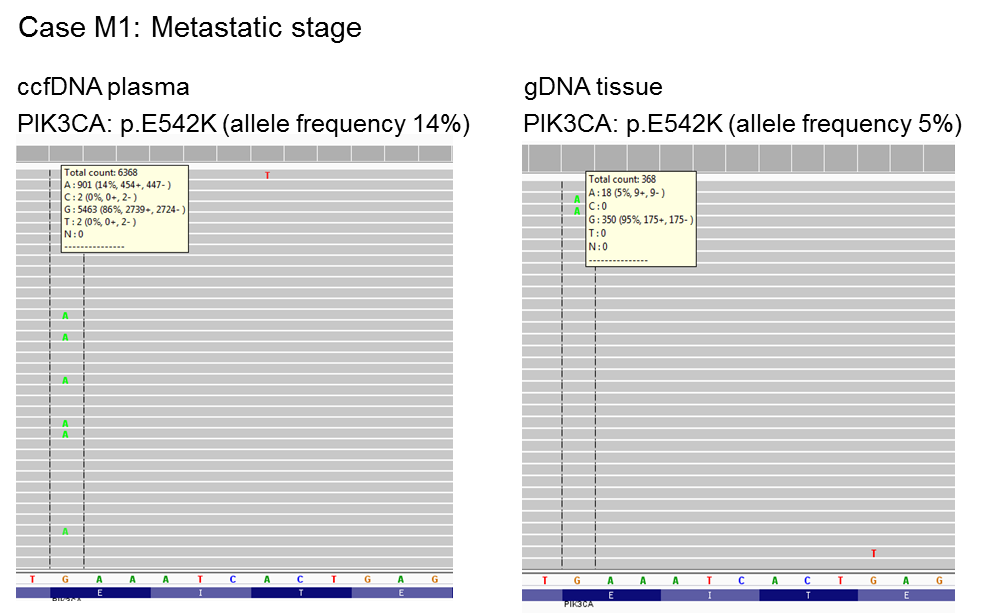


**Supplemental Figure SF1.** Next generation sequencing (NGS) for case M1.

| **Case** | **Staging on resected tumours** | **Treatment** |
| --- | --- | --- |
| 1 | ypT2,ypN1 (1/27),L1,V0,R0 | laparoscopic gastrolysis, transthoracic en bloc oesophagectomy including two-field lymphadenectomy, reconstruction by intrathoracic oesophagogastrostomy (Ivor Lewis) |
| 2 | n.a. (no resection) | definitive radio-chemotherapy, no surgery (aortic stenosis) |
| 3 | pT1a (M3), pN00, R0 | laparoscopic gastrolysis, transthoracic en bloc oesophagectomy including two-field lymphadenectomy, reconstruction by intrathoracic oesophagogastrostomy (Ivor Lewis) |
| 4 | ypT1a(m3), ypN0(0/24), L0, V0, R0 | laparoscopic gastrolysis, transthoracic en bloc oesophagectomy including two-field lymphadenectomy, reconstruction by intrathoracic oesophagogastrostomy (Ivor Lewis) |
| 5 | n.a. (no resection) | definitive radio-chemotherapy, no surgery (high reaching tumour localization) |
| 6 | pT3, pN0 (0/47), L0, V0, pn1, R0 | total gastrectomy with inferior oesophagectomy |
| 7 | n.a. (no resection) | definitive radio-chemotherapy, no surgery |
| 8 | ypT3, ypN1 (2/12), L1, V1, pn0, R0 | total gastrectomy with inferior oesophagectomy |
| 9 | pT1a, pN0(0/31), V0, L0, R0 | laparoscopic gastrolysis, transthoracic en bloc oesophagectomy including two-field lymphadenectomy, reconstruction by intrathoracic oesophagogastrostomy (Ivor Lewis) |
| 10 | n.a. (no resection) | definitive radio-chemotherapy, no surgery (aortic aneurysm) |
| 11 | ypT3, ypN0(49), L0, V0, R0 | laparoscopic gastrolysis, transthoracic en bloc oesophagectomy including two-field lymphadenectomy, reconstruction by intrathoracic oesophagogastrostomy (Ivor Lewis) |
| 12 | n.a. (no resection) | definitive radio-chemotherapy, no surgery (patient's wish) |
| 13 | ypT1b (SM2), ypN1 (1/35), V0, L0, R0 | laparoscopic gastrolysis, transthoracic en bloc oesophagectomy including two-field lymphadenectomy, reconstruction by intrathoracic oesophagogastrostomy (Ivor Lewis) |
| 14 | pT1b, pN1 (1/35), L1, V0, local R0 | laparoscopic gastrolysis, transthoracic en bloc oesophagectomy including two-field lymphadenectomy, reconstruction by intrathoracic oesophagogastrostomy (Ivor Lewis) |
| 15 | uT3, ypT0 ypN0 (0/24) L0 V0 R0 | gastrolysis, transthoracic en bloc oesophagectomy including two-field lymphadenectomy, reconstruction by intrathoracic oesophagogastrostomy (Ivor Lewis) |
| 16 | ypT3, ypN0 (0/15), L0, V0, R0 | laparoscopic gastrolysis, transthoracic en bloc oesophagectomy including two-field lymphadenectomy, reconstruction by intrathoracic oesophagogastrostomy (Ivor Lewis) |
| 17 | pT1b, pN0 (0/53), V0, L0, R0 | subtotal D2-gastrectomy |
| 18 | pT4a, pN0 (0/17), V0, L0, R0 | subtotal D2-gastrectomy |
| 19 | ypT0, pN0 (0/32), L0, V0, R0 | laparoscopic gastrolysis, transthoracic en bloc oesophagectomy including two-field lymphadenectomy, reconstruction by intrathoracic oesophagogastrostomy (Ivor Lewis) |
| 20 | pT1a, pN0 (0/31), L0, V0, local R0 | laparoscopic gastrolysis, transthoracic en bloc oesophagectomy including two-field lymphadenectomy, reconstruction by intrathoracic oesophagogastrostomy (Ivor Lewis) |
| 21 | ypT3, ypN3 (8/19), L0, V0, R0 | laparoscopic gastrolysis, transthoracic en bloc oesophagectomy including two-field lymphadenectomy, reconstruction by intrathoracic oesophagogastrostomy (Ivor Lewis) |
| 22 | pT3, pN2 (4/26), L0, V0, R0 | laparoscopic gastrolysis, transthoracic en bloc oesophagectomy including two-field lymphadenectomy, reconstruction by intrathoracic oesophagogastrostomy (Ivor Lewis) |
| 23 | pT3, pN3 (9/33), L1, V0, R0 | laparoscopic gastrolysis, transthoracic en bloc oesophagectomy including two-field lymphadenectomy, reconstruction by intrathoracic oesophagogastrostomy (Ivor Lewis) |
| 24 | n.a. (no resection) | definitive radio-chemotherapy, no surgery (surgery not completed, cirrhosis of the liver) |
| 25 | ypT0, ypN0 (0/18), L0, V0, R0 | laparoscopic gastrolysis, transthoracic en bloc oesophagectomy including two-field lymphadenectomy, reconstruction by intrathoracic oesophagogastrostomy (Ivor Lewis) |
| 26 | pT1b, pN0 /0/26), L0, V0, local R0 | laparoscopic gastrolysis, transthoracic en bloc oesophagectomy including two-field lymphadenectomy, reconstruction by intrathoracic oesophagogastrostomy (Ivor Lewis) |
| 27 | ypT3, pN2 (4/33), L1, V0, R0 | laparoscopic gastrolysis, transthoracic en bloc oesophagectomy including two-field lymphadenectomy, reconstruction by intrathoracic oesophagogastrostomy (Ivor Lewis) |

**Supplemental Table S1.** Applied treatments and pathological staging of all patients included within the current study.

| **Gene** | **Exons** | **Codons** |
| --- | --- | --- |
| BRAF | 15 | 582-612 |
| DDR2 | 3-18 | 1-342, 351-856 |
| ERBB2 | 8, 19-21 | 302-321, 980-1069, 761-881 |
| HRAS | 2-4 | 1-35, 41-82, 105-150 |
| KEAP1 | 2-6 | 1-34, 37-122, 127-437, 443-625 |
| KRAS | 2-4 | 4-66, 111-150 |
| NFE2L2 | 2 | 16-104 |
| NRAS | 2-4 | 1-32, 45-83, 110-150 |
| PIK3CA | 1, 4, 7, 9, 20 | 66-117, 312-350, 418-435, 521-554, 980-1069 |
| PTEN | 1-9 | 1-34, 39-267, 275-333, 343-366 |
| RHOA | 2, 3 | 3-89 |
| TP53 | 5-9 | 133-260, 263-331 |

**Supplemental Table S2.** Ion AmpliSeq™ Custom DNA Panel

| **Gene** | **Mutation** | **dHSA Nummer (Bio-Rad Website)** |
| --- | --- | --- |
| TP53 | p.L145Rfs*25 | MDS116079217 (custom made) |
| TP53 | p.F134S | MDS466710389 (custom made) |
| TP53 | p.G245A | MDS108910884 (custom made) |
| TP53 | p.G245S | CP2506746 |
| TP53 | p.P278L | CP2506856 |
| TP53 | p.R248W | CP2000107 |
| TP53 | p.R273H | CP2000109 |
| TP53 | p.R282W | CP2506902 |
| TP53 | wildtype | CP2000110 |
| TP53 | p.R248Q | MDV 2010127 (custom made) |
| TP53 | p.Y205S | MDS2516070 (custom made) |
| TP53 | p.H193P | MDS2514438 (custom made) |
| TP53 | c.559+1G>A | MDS143739847 (custom made) |
| TP53 | c.376-3C>G | MDS765323428 (custom made) |
| ERBBB2 | p.G776V | MDS497748569 (custom made) |
| KRAS | p.G12A | CP2500586 |
| KRAS | wildtype | CP2500587 |
| BRAF | p.D594G | MDS983054417 (custom made) |
| PIK3CA | p.Q546P | MDS487077669 (custom made) |
| PIK3CA | p.E542K | CP2000073 |
| PIK3CA | p.E542K_wt | CP2000074 |
| PIK3CA | p.E545K | CP2000075 |
| PIK3CA | p.E545K_wt | CP2000076 |

**Supplemental Table S3.** Order numbers and ddPCR
